# Why Do Farmers Grow Tobacco? A Qualitative Exploration of Farmers Perspectives in Indonesia and Philippines

**DOI:** 10.3390/ijerph16132330

**Published:** 2019-07-02

**Authors:** Adriana Appau, Jeffrey Drope, Firman Witoelar, Jenina Joy Chavez, Raphael Lencucha

**Affiliations:** 1 Faculty of Medicine, School of Physical and Occupational Therapy, McGill University, 3630 Promenade Sir William Osler, Montreal, QC H3G 1Y5, Canada; 2 Economic and Health Policy Research, American Cancer Society, Atlanta, GA 30303, USA; 3 Crawford School of Public Policy, Australian National University, Canberra, ACT 2601, Australia; 4 Action for Economic Reform, Unit 1403 West Trade Center, 132 West Avenue, Quezon City 1104, Philippines

**Keywords:** tobacco control, tobacco farming, alternative livelihood, qualitative analysis

## Abstract

Tobacco supply remains a pressing challenge to tobacco control. Tobacco remains a dominant cash crop in many low- and middle-income countries, despite the evidence suggesting that it is not as profitable as industry claims and is harmful to health and the environment. In order to implement successful and sustainable alternative livelihood interventions, it is important to understand why farmers continue to grow tobacco. This study explores this question from the perspective of farmers in Indonesia and Philippines. This study was informed by interpretive description methodology. Data was collected through focus group discussions (FGDs) (*n* = 7) with farmers (*n* = ~60). The FGDs were audio recorded, transcribed verbatim, and then translated into English. An inductive thematic analysis of the data was conducted to identify and categorize the reason provided by participants. We identified two overarching themes: (1) perceived viability (profitability, ready market, and environmental factors) and (2) financial context. Financial context included lumpsum payments and access to financial loans and credit facilities in light of their lack of capital. These results highlight that, in addition to identifying viable alternatives to tobacco, institutional factors such as improved access to credit and well-developed supply chains are key to the successful uptake of alternative livelihoods.

## 1. Introduction 

Tobacco use is the largest preventable risk factor for most major non-communicable diseases [1]. The consumption of tobacco products requires one major input: tobacco leaf. Despite having important implications for public health, the supply-side dimensions of tobacco control receive little scholarly attention. Though important scholarship on the economic complexities of growing tobacco has evolved, particularly about the experience of the hundreds of thousands of smallholder farmers who supply most of the world’s tobacco leaf, there is much work to be done [2,3,4,5]. The public health treaty that frames global tobacco control, the World Health Organization Framework Convention on Tobacco Control, recognizes the importance of tobacco production and includes Article 17, which obligates Parties to help tobacco farmers and others employed in the tobacco sector to find viable alternative economic livelihoods. To inform this treaty provision, and particularly to identify pathways out of tobacco growing, there is much to gain from tobacco farmers’ perspectives.

The tobacco industry has long perpetuated a narrative of tobacco farming as economically prosperous, despite evidence to contrary [6,7,8,9,10,11,12,13]. Recent farm-level research across at least five countries utilizing household economic surveys systematically illustrates the opposite scenario: most smallholder farmers endure economic loss [9,10,14,15,16], which is often significant and consistent over many years. Yet, despite overwhelming evidence of economic struggle, farming remains thoroughly entrenched in many mostly lower-income countries. Institutional forces shape a considerable part of the persistence of tobacco growing, including governments that provide incentives to produce [17,18]. Similarly, related research demonstrates that many governments continue to focus on the macro-level perceived economic benefits of tobacco such as foreign exchange generation and export potential, often with a limited understanding for the livelihood realities of the individual tobacco farmers and their households [18].

While the institutional context undoubtedly affects farmers’ decisions to grow tobacco, decisions made at the household level also shape what farmers grow. Considering the economic struggle of smallholder farmers in most low-income countries, above all else, these farmers are arguably searching most for livelihoods that they believe will be viable for them. For example, farmers typically have some knowledge of the prices paid for crops in previous seasons and compare it to prices of other crops, including ones that they have grown or are currently cultivating [19]. Part of this broader economic calculation is also the ease of obtaining credit to grow a specific crop, which is a near necessity due to the micro-economic cycle of farmers requiring many inputs at the start of a growing season long before production generates revenue [20]. Similarly, farmers look for crops that generate cash in an economic context of near subsistence where cash is often scarce [21]. Not surprisingly, farmers must also consider market opportunities, and particularly the ease of selling their crops [22,23]. Even in high-income countries, similar factors including lack of an existing market for alternative crops and absence of low-interest loans to support new ventures have been found to shape decisions to continue growing tobacco [24]. Finally, farmers are typically aware of which crops have been previously grown—successfully and unsuccessfully—in their locality based on soil, climate/weather, and other environmental conditions [19]. There is also a social dimension to farming. Farmers often report “inheriting” tobacco growing from their parents [16,25]. Similarly, in communities where tobacco farming is widespread, there may be implicit social pressures to grow tobacco both because the structures exist for it (such as auction houses) but also because almost everyone is somehow involved in its production and may have been for generations, often seemingly regardless of the economic reward.

Considering these dynamics, we examine farmers’ perspectives in Indonesia and the Philippines, two major tobacco-growing countries in Southeast Asia. We chose these countries because tobacco farming is not only robust in several major agricultural regions, but also because tobacco growing continues to be used as a reason by the governments not to implement effective tobacco control policies, including increasing excise taxes. Their explicit logic is that tobacco control will hurt the livelihoods of these farmers even though recent research demonstrates that tobacco is a losing economic proposition for most farmers in both countries. The following two research questions guided this study: (1) what reasons do tobacco farmers provide for why they grow tobacco and (2) what factors do they identify that might lead them to stop growing tobacco?

## 2. Materials and Methods

Our study was informed by an interpretive description methodology that aims to generate practical knowledge and “offers the potential to deconstruct the angle of vision upon which prior knowledge has been erected and to generate new insights that shape new inquiries” [26]. Interpretative description views social reality as contextual and subjective, pointing to the importance of gaining first-person accounts of how individuals view their reality [26]. Our own analysis is informed by interpretative description, whereby we aim to identify and present common patterns and differences from the perspective of farmers [27]. We aimed to gain new insights into why farmers choose to grow tobacco and place emphasis on farmers’ perceptions and perspectives [27]. Although there is some literature, mainly based on quantitative studies, on why farmers grow tobacco, there is limited research that foregrounds the perspectives of farmers. This perspective is particularly important given that, for example, early quantitative studies have found that tobacco farming may be less profitable and more labour intensive than other crops. Yet, we know little about why farmers would continue to grow a crop under these unfavourable conditions.

Data was collected using focus group discussions (FGD) (*n* = 7) with farmers who grow tobacco (*n* = ~60). The FGD was chosen because it allows one to obtain a broader range of perspectives and insights on a particular topic compared to individual interviews. It is also useful in situations when participants share common practices in a shared context [28], in this case growing tobacco in a particular region. The interactive nature of this method allows for participants to build on shared experiences through emergent discussion [29]. Furthermore, there is an added advantage of identifying a group perspective on a particular issue [29]. We also aimed to encourage different perspectives within the group rather than requiring consensus or agreement. The FGDs were conducted by members of the research team, with professional FGD facilitators indigenous to each community using a common interview guide. Recruitment for the FGDs was based on a purposive sample. Focus group discussions were conducted in villages chosen purposively in each country’s major tobacco-growing area. In Indonesia, in the first three months of 2017, we conducted FGDs in three municipalities: Sugigwaras (Alasagung), Ngraho (Nganti), and Ngasum. In the village of Alasagung and Nganti, we conducted FGDs with active tobacco farmers. In the village of Mediunan, we conducted FGDs with three discrete groups: current tobacco farmers, former tobacco farmers, and the middlemen who buy tobacco from farmers and sell it to the tobacco processors and/or manufacturers. In the Philippines, the team conducted three FGDs in July 2015 with current tobacco farmers from each of Illocos Sur and La Union provinces on Luzon and the farming community immediately surrounding Cagayan del Oro on Mindanao. Local leaders in each farming community extended an invitation to current and former tobacco farmers to participate in the FGDs. A list of farmers who were interested in participating was provided to the research team who then contacted eligible farmers. To be eligible, the farmer must be a current or former tobacco farmer and not a village official or head. The survey team supervisor that was responsible in the study area grouped the participants for each FGD. Each group had between 8 and 12 participants. All discussions were conducted in the local language or dialect, audio recorded, transcribed verbatim, and translated into English, and then the translation was subjected to quality assurance from a bilingual native speaker. The facilitators were local to the area in order to facilitate comfort and cultural alignment in the communication. One researcher from the core team attended each focus group. The facilitators kept detailed notes on the seating arrangement, and participation level, of each participant. 

The data were coded inductively to identify common reasons for growing tobacco and differences among participants. First, all transcripts were read thoroughly. This was followed by a line-by-line analysis of each transcript to identify reasons why farmers grow tobacco. The transcripts were read for a third time to group similar data into broader categories [30]. The lead author and RL met to review the initial categories, the data that populated them, and dialogue; the transcripts generated a final set of categories that seemed to best answer the research question. During this process, relationships between categories and sub-categories were also identified. All analysis was conducted using NVivo 12 software (QSR International Pty Ltd, Doncaster, Victoria, Australia). Ethics approval for this study was obtained from the Institutional Review Boards of the Morehouse School of Medicine (the IRB record for the American Cancer Society), the government of the Philippines, and SurveyMeter (Indonesia).

## 3. Results

We clustered the findings into two categories that represented the main reasons why farmers grow tobacco: perceived viability and financial context. These two categories are populated by five sub-categories, as shown in Table 1.

### 3.1. Perceived Viability

Participants expressed that tobacco was the only viable crop and felt that there was a lack of alternatives to tobacco growing. We found that viability was characterized by three sub-categories: profitability, availability of market, and nature of tobacco.

#### 3.1.1. Profitability

Participants repeatedly expressed that tobacco was profitable compared to other crops, because it was the highest income earner for their families. The notion of profit was conceptualized differently by participants, ranging from total income to the ability to purchase items. Despite the differences, all participants compared the profitability of tobacco with other crops, not other employment opportunities. This is illustrated by one participant who stated that *“The other crops can only give us a minimal income”* (Philippines). Another participant from Indonesia expressed that *“Besides tobacco … the last 2 years I planted chili, but suffering from financial loss”* (Indonesia). For other farmers, they perceived tobacco to be profitable because they were able to meet their needs with income generated from selling tobacco leaf. For example, when asked if tobacco was profitable one farmer said *“Yes, it is profitable. We can buy what we want, we were able to repair our house”* (Philippines). This notion of tobacco being profitable, however, was not shared by all participants. For some participants the profitability of tobacco was not consistent, making it difficult to meet needs from year to year:

In ten years, 5 years we lose, 5 years we gain. In tobacco farming, it’s not like you will prosper, no. You just earn for your everyday needs, you don’t earn so much and become a millionaire. What we earn is just for everyday sustenance. If there is something left, it’s not enough.

(Philippines)

Furthermore, it is worth noting that this “perceived profitability” did not account for labour cost. Some farmers who said tobacco was profitable expressed that they intentionally did not account for their labour because they said *“We are too ashamed to include ourselves, you know… Also because when you include them, you might just be depleting the profit”* (Philippines). Similarly in Indonesia, when farmers were asked if they based their decisions on growing tobacco on previous years revenue most of them were of the view that *“No matter what, I keep growing tobacco… No matter what, keep growing. If I (am) lucky I will get profit”* (Indonesia). Whether tobacco farming was profitable in the previous years or not, the farmers expressed willingness to continue growing tobacco in anticipation of the next profitable year.

#### 3.1.2. Availability of Market

Tobacco farmers can choose to farm under a contract with a tobacco company or as an independent farmer. Farming under contract guaranteed a buyer for tobacco leaf at the end of the season. Having a ready market for their tobacco leaf was one of the main reasons why farmers continued to grow tobacco. For instance, when asked if any of the participants from Indonesia had considered replacing tobacco with other crops, one participant responded *“As long as there is a partnership, still tobacco”* (Indonesia), as these partnerships guaranteed them a market. Furthermore, one participant in reference to alternative crops suggested *“There should be a buyer also with a contract”* (Philippines). The absence of similar market linkages for other crops was a major deterrent from switching to alternative crops in light of the ready market for tobacco. Most participants were of the view that it was often difficult to find buyers for other crops. As one participant explained: *“You have to scout for buyers, unlike in tobacco that regardless of the amount you want to sell, there is always a market”* (Philippines).

In addition to having a ready market, participants explained that even in circumstances where the grade of the tobacco was low they were still able to sell it to get some income. As one participant noted, *“even if a storm hits us, we will still be able to sell our tobacco”* (Philippines). They noted that when other crops receive a lower grade, the harvest is rejected. In effect, they continue to grow tobacco because they are certain of receiving some income even if it is lower than expected.

#### 3.1.3. Environmental Factors

Viability seemed to also be tied to the relationship between the tobacco leaf and the natural environment. Participants expressed that environmental factors, associated with the resilience of tobacco to adverse weather conditions compared to other crops, shaped their decisions to grow tobacco. The perception was that, although most crops may be affected by droughts or excessive rain, they believe tobacco is more resilient, allowing them to still make some income at the end of the growing season. One farmer explained that

Tobacco can grow during hottest period, likewise when there is rain. With enough fertilizer, tobacco can grow and produce good quality of leaves. Even the small branches can produce leaves, and these are additional income. That’s why tobacco is better than corn. In the case of corn, once the leaves wither, it can no longer be saved, it will eventually die. That’s the reason why we will never give-up tobacco.

(Philippines)

In some regions, environmental factors such as land topography, soil type, and access to water for irrigation were given as reasons why farmers were growing tobacco. For example, in one of the regions in Indonesia, participants indicated that the soil was not conducive for growing any other crops. He stated

“I have said before; the land is only suitable for tobacco and marijuana”.

(Indonesia)

### 3.2. Financial Context 

Participants also noted that tobacco provided financial advantages separate from income or profits. Participants noted that access to capital and inputs was one of the key reasons they grew tobacco.

#### 3.2.1. Access to Financial Loans and Lack of Capital

Participants noted that they lacked the needed capital to grow tobacco or any other crops. They therefore needed loans to be able to grow crops. However, with the uncertainty in crop yields and the risk of not being able to repay loans, some farmers were unable to access loans from financial institutions. One farmer shared: *“For example my rice in the field looks so good but then there is pest infestation when the time to harvest come then farmers have no money to pay back the loan, so we are not brave enough to apply for loan in a bank”* (Indonesia). In connection to this, a farmer said, *“The middleman provides loan with no interest, we pay the price based on the market price”* (Indonesia), thus farming tobacco gives them access to loans with favorable terms of payment. Similarly, in the Philippines, the tobacco industry (tobacco buying companies, independent agents) provided farmers with financial loans before the tobacco growing season and in some cases outside the growing season. These loans were used both as initial capital for tobacco farming and also for non-agricultural expenses such as paying school fees, buying food, and payment of other loans. Part of these loans were also used to buy inputs (e.g., seeds, fertilizer) for growing food crops such as corn and vegetables. For instance, when farmers were asked how important tobacco farming was to them one answered *“Despite the hardship in planting tobacco, I will still plant tobacco. If I stop I will not be able to borrow money”* (Philippines). Another participant noted that *“If ever we borrow money mam, it is not used exclusively for tobacco only. So if our other crops like vegetables or corn also need fertilizer, we use the money for that purposes”* (Philippines). 

There was great emphasis on the importance of receiving these financial loans to their farming activities. According to participants especially in the Philippines, they would not have access to these loans if they cultivated other crops and not tobacco. One participant from the Philippines noted that *“For other crops, resources are limited. You cannot borrow if you plant peanuts, for example, and no one will lend you seeds and fertilizer. Unlike in tobacco, you can proceed planting even if you don’t have the needed capital.”* (Philippines)

Participants expressed a loyalty to the tobacco companies because of the provision of financial benefits such as health insurance coverage and materials such as equipment such as water pumps, rain coats and boots for free. For instance, when participants were asked if they plant other crops, one farmers said “*I grow only tobacco…We are loyal to tobacco*” (Philippines). When asked if they receive incentives the farmers shared the following: *“They give incentives at a time of difficulty”* (Philippines). One farmer then added that *“That’s one reason why we are loyal to the company”* (Philippines).

#### 3.2.2. Lump-Sum Accumulated Savings

The participants stated that in most cases they receive a lump sum payment for the tobacco harvested in the year. All of the farmers noted that this was an important aspect of tobacco growing. The farmers viewed the lump sum payment as a reward for their hard work, noting that tobacco farming is labour intensive, requires more inputs, and is physically demanding. They noted the satisfaction from receiving a large cash payment in hand at the end of the growing season. As two participants noted

As long as we have sent it to (company), then going home with money is already called refreshing.

(Indonesia)

That’s why we also like planting tobacco, when you deliver tobacco you handle huge amount of money. As if it’s yours. More so if it’s a good harvest and the price is good, that would make one happy.

(Philippines)

In addition, participants noted that the lump sum payment served as an involuntary saving mechanism as explained by the following participant from Indonesia: *“It gives us a good feeling once we have sold our products Because when we sell, the money is whole and intact. Last year, for example, my gross sales were P200,000, that was intact cold cash”* (Indonesia). Another participant likened the lump sum payment to *“a piggy bank”* (Indonesia). These involuntary savings allowed them to undertake large financial projects like building houses after receiving this lump sum payment as indicated by this participant: *“We can buy what we want. We were able to repair our house. It has helped me”* and *“I was able to buy a motorcycle for service”* (Philippines).

Although the participants seemed happy to receive lump sum payments and believed tobacco farming was profitable, they seem to suggest an illusory dimension to receiving this payment, as they made statements like “*All the money seems yours*” (Philippines). Many participants noted that most of the money is used to pay off loans, debts, and hired labour, leaving a small portion of the “huge amount” as profit.

Participant 6: We get excited when we deliver tobacco. A 3-hectare yield will bring huge profit. Participant 4: All the money seems yours

Participant 6: It’s like the money is all yours, but only for a while because you need to pay… The following day the money is all gone, you’ve used all the money. One day millionaire.

(Philippines)

## 4. Discussion 

Tobacco farming has negative health, environmental, and economic consequences [31,32]. Despite the negative aspects of tobacco growing, and attempts to implement interventions promoting alternative livelihoods, many farmers continue to grow tobacco. This qualitative study provides insights into the reasons why farmers continue to grow tobacco in the Philippines and Indonesia. Farmers were of the view that tobacco is the only viable crop because of its perceived high profits, available markets, and resilience to adverse weather conditions. In addition, they continue to grow tobacco because they believe it allows them to access financial and input loans in a context where they lack the needed capital for farming. Furthermore, participants expressed that lumpsum payment from tobacco sales served as unintended savings to undertake financial projects, such as building and renovating their homes.

We found that participants explicitly tied the viability of tobacco to the environmental conditions, suggesting that tobacco was best suited to the growing environment. Similar results were obtained in a recent qualitative study on reasons why farmers grow tobacco in India [19]. According to their analysis, farmers grew tobacco because of its resilient nature to droughts and the high earnings obtained from tobacco compared to other crops [19]. However, it should be noted that within these FGDs, participants consistently noted that they grew other crops. Other studies in Indonesia have also found that farmers allocate less than half of their land to tobacco cultivation [15]. This suggests that the environmental conditions did not limit them to tobacco, and perhaps it was economic conditions that largely dictated the decision to grow tobacco. 

Supply chain management is an integral part of agricultural production, including access to markets. Farmers repeatedly identified this dynamic as crucial to their decision to cultivate tobacco. This was also the reason for the popularity of contract farming, even though it typically provided unfair terms and poor returns [18,21]. Contract farming is a way for companies to meet production needs and quality of crops, but also theoretically assures a buyer for farmers’ products [33]. Some studies have found that contract farming increases production efficiency and farm incomes, and serves as a form of insurance against price fluctuations [34,35,36]. The expressed logic of contracts is also to manage production, by providing extension services to farmers and by standardizing the provision of inputs such as fertilizer and pesticide [33]. However, there is a complex political economy surrounding contract farming that tempers these intended benefits. For instance, there is evidence to support that contract farmers pay higherthan-market prices for inputs received from the industry and are often not satisfied with the grade assigned and price obtained at the time of selling their tobacco leaf [9,15].

The perceived lack of the readily available market for alternative crops was a major concern raised by farmers, who had indicated they were not willing to cultivate other crops. Similar concerns were raised by tobacco farmers in the United States in the 1990’s about the lack of processing factories to connect farmers to potential consumers [37]. Interventions that identify and link buyers who form equitable partnerships with farmers have the potential to increase farmers productivity and quality of alternative crops [38]. There is some research to suggest that collective action through smallholder farmer cooperatives or producer organizations helps smallholder farmers overcome market barriers such as high transportation costs and gain better access to financial services and resources [39,40,41].

The farmers in these FGDs consistently identified a lack of capital and limited access to credit facilities and input markets to be major barriers to switching to alternative crops. Participants noted that they lacked the ability to raise capital for growing both tobacco and other crops at the beginning of the growing season. Furthermore, they suggested that they had little to no access to formal credit facilities outside the tobacco industry to cover both agricultural and non-agricultural expenses such as paying for children’s education. This situation is found in other settings and with other crops [40,42,43,44]. For example, in a Food and Agriculture Organization of the United Nations report on the economic livelihoods of smallholder farmers in nine developing countries [45], the majority of famers did not have access to formal credit, which affected productivity and household income. In some of the countries, farmers had to sell their products at lower prices to be able to raise money to pay non-agricultural expenses [45]. The tobacco industry has created an attractive system for smallholder farmers by providing capital and financial and input loans through the contract process. This dimension of the supply chain has also been shown to feature prominently in farmer decisions to grow tobacco in other countries [20]. As indicated by most farmers in this study, these loans are sometimes obtained well in advance of the growing season and often also used to cultivate non-tobacco crops and pay off non-agricultural expenses such as education. Improving access to capital and credit facilities may be one viable way to increase uptake of alternative crops and improve farm incomes [40]. 

Provision of technical support, including financial and accounting training, has the potential to increase the likelihood and viability of switching to alternative livelihoods. Our findings suggest that some farmers indicated that non-tobacco products are not as profitable as tobacco. However, as admitted by some of the participants, they do not include any value for family labour in their calculation of profits. However, tobacco farming is both labour- and input-intensive. Proper accounting of the labour and input requirements of tobacco growing suggests that it may not be as profitable as perceived by farmers [9,46]. In fact, in Indonesia, the average income of former tobacco farmers was higher than that of current tobacco farmers [15]. Not only did they grow other crops for profit, but they reallocated their time for other economically productive off-farm activities (such as paid work, small transport businesses, and small-scale retailing). If farmers are well trained to calculate their input costs, it will likely influence agricultural decisions such as crop choice and the optimal use and allocation of their farm resources, including labour. In addition, training programs that provide the necessary skills to grow other crops, especially for older and less educated farmers, will ease their transition into alternative livelihoods [47].

This study also has highlighted an interesting social dimension in the tobacco growing context. Participants expressed that their relationship to the tobacco company and the crop itself was one reason why they continued to grow tobacco. In our study, especially in the Philippines, loyalty to tobacco or the tobacco industry seemed to stem from farmers’ appreciation for the industry’s long-term support in providing loans that meet their daily needs, extension services, provision of rewards and incentives such as price adjustments, and paying for their health insurance and farm equipment. Many of the farmers expressed how they could rely on tobacco and the tobacco industry in times of need, whether it was associated with tobacco farming or not. We did not necessarily find that loyalty was associated with farmer’s satisfaction, as there was a general sense of dissatisfaction with tobacco prices and leaf quality grading; the labour requirements; and, for some, tobacco revenue. Rather, they seemed to value their relationship with the industry, with some referring to them as “family” and the achievement they have made through tobacco farming, implying a more personal attachment to tobacco farming. Extending extra non-contractual benefits and, more importantly, the relationship between the firm and farmers, also observed in other studies, was more influential in maintaining effective contractual agreements [48].

This study has some limitations. First, although experienced researchers facilitated the FGDs, it is possible that those who were most vocal took over the conversation and that the experiences and views of these individuals did not reflect the views of the group. Despite these possible limitations, the FGD facilitator worked to ensure that all participants expressed their perspective during the process. Second, follow-up FGDs may have added more details to clarify some of the factors identified during data analysis. For example, follow up may have helped to illustrate the nature of the relationship between farmers and tobacco companies, or specific experiences growing other crops. Third, it is possible that some meaning was lost in the translation from the local languages to English. However, given the descriptive nature of the analysis, we think that the perspectives presented for the most part reflect the intended meaning.

## 5. Conclusions

Tobacco leaf supply remains a challenge to tobacco control efforts to reduce tobacco consumption globally. At the center of this is the perpetuation of tobacco farming, especially in low- and middle-income countries. This study identifies that tobacco farmers continue to grow tobacco, amongst other things, because of its perceived profitability, resilience to adverse weather, the availability of an existing market for tobacco, and access to credit. It is important that proponents of alternative livelihoods address the factors that contribute to farmer’s decisions to continue growing tobacco. For instance, policies aimed at promoting alternative crops need to identify crops that are environmentally viable for cultivation. Institutional factors such as improved access to credit and well-developed supply chains are key to the successful uptake of alternative livelihoods in farming.

## Figures and Tables

**Table 1:** Findings.

Category	Sub-Category	Representative Quotes
Perceived Viability	Profitability	*“The other crops can only give us a minimal income”*
	Availability of Market	*“You have to scout for buyers, unlike in tobacco that regardless of the amount you want to sell, there is always a market”*
	Environmental Factors	*“I have said before; the land is only suitable for tobacco and marijuana”*
Financial Context	Access to Financial Loans and Lack of Capital	*“Despite the hardship in planting tobacco, I will still plant tobacco. If I stop, I will not be able to borrow money”*
	Lump-Sum Accumulated Savings	*“As long as we have sent it to (company), then going home with money is already called refreshing”*

## References

[1] Reitsma M.B., Fullman N., Ng M., Salama J.S., Abajobir A., Abate K.H., Abbafati C., Abera S.F., Abraham B., Abyu G.Y. (2017). Smoking prevalence and attributable disease burden in 195 countries and territories, 1990–2015: A systematic analysis from the Global Burden of Disease Study 2015. Lancet.

[2] Lecours N. (2014). The Harsh Realities of Tobacco Farming: A Review of Socioeconomic, Health and Environmental Impacts. Tobacco Control and Tobacco Farming: Separating Myth from Reality.

[3] Otanez M.G., Mamudu H., Glantz S.A. (2007). Global leaf companies control the tobacco market in Malawi. Tob. Control.

[4] Prowse M. (2013). A history of tobacco production and marketing in Malawi, 1890–2010. J. East. Afr. Stud..

[5] Moyer-Lee J., Prowse M. (2015). How Traceability is Restructuring Malawi’s Tobacco Industry. Dev. Policy Rev..

[6] Warner K.E. (2000). The economics of tobacco: Myths and realities. Tob. Control.

[7] Van Minh H., Giang K.B., Bich N.N., Huong N.T. (2009). Tobacco farming in rural Vietnam: Questionable economic gain but evident health risks. BMC Public Health.

[8] Leppan W., Lecours N., Buckles D. (2014). Tobacco Control and Tobacco Farming: Separating Myth from Reality.

[9] Makoka D., Drope J., Appau A., Labonte R., Li Q., Goma F., Zulu R., Magati P., Lencucha R. (2017). Costs, revenues and profits: An economic analysis of smallholder tobacco farmer livelihoods in Malawi. Tob. Control.

[10] Magati P., Lencucha R., Li Q., Drope J., Labonte R., Appau A.B., Makoka D., Goma F., Zulu R. (2019). Costs, contracts and the narrative of prosperity: An economic analysis of smallholder tobacco farming livelihoods in Kenya. Tob. Control.

[11] Gilmore A.B., Fooks G., Drope J., Bialous S.A., Jackson R.R. (2015). Exposing and addressing tobacco industry conduct in low-income and middle-income countries. Lancet.

[12] Otañez M.G., Mamudu H.M., Glantz S.A. (2009). Tobacco Companies’ Use of Developing Countries’ Economic Reliance on Tobacco to Lobby Against Global Tobacco Control: The Case of Malawi. Am. J. Public Health.

[13] WHO Framework Convention on Tobacco Control (2014). Economically Sustainable Alternatives to Tobacco Growing (in Relation to Articles 17 and 18 of the WHO Framework Convention on Tobacco Control).

[14] Chavez J., Drope J., Aloria M. (2016). The Economics of Tobacco Farming in the Philippines.

[15] Drope J., Li Q., Araujo E., Harimurti P., Sahadewo G., Nargis N., Durazo J., Witoelar F., Sikoki B. (2017). The Economics of Tobacco Farming in Indonesia.

[16] Goma F., Drope J., Zulu R., Li Q., Chelwa G., Labonte R., Banda J. (2016). The Economics of Tobacco Farming in Zambia.

[17] Lencucha R., Drope J., Labonte R., Zulu R., Goma F. (2016). Investment incentives and the implementation of the Framework Convention on Tobacco Control: Evidence from Zambia. Tob. Control.

[18] Labonté R., Lencucha R., Drope J., Packer C., Goma F.M., Zulu R. (2018). The institutional context of tobacco production in Zambia. Glob. Health.

[19] Natarajan N. (2018). Moving past the problematisation of tobacco farming: Insights from South India. Tob. Control.

[20] Ochola D.S.A., Kosura P.W. (2017). Case Study on Tobacco Cultivation and Possible Alternative Crops—Kenya.

[21] Niño H.P. (2016). Class dynamics in contract farming: The case of tobacco production in Mozambique. Third World Q..

[22] Lukanu G., Green M., Greenfield P., Worth S. (2004). Farmers’ cash crop cultivation decisions in Southern Niassa province, Mozambique. Dev. South. Afr..

[23] Greig L. (2009). An Analysis of the Key Factors Influencing Farmer’s Choice of Crop, Kibamba Ward, Tanzania. J. Agric. Econ..

[24] Altman D.G., Zaccaro D.J., Levine D.W., Austin D., Woodell C., Bailey B., Sligh M., Cohn G., Dunn J. (1998). Predictors of crop diversification: A survey of tobacco farmers in North Carolina (USA). Tob. Control.

[25] Makoka D., Drope J., Appau A., Lencucha R. (2016). Farm-Level Economics of Tobacco Production in Malawi.

[26] Thorne S.E. (2008). Interpretive Description (Developing Qualitative Inquiry).

[27] Thorne S., Kirkham S.R., O’Flynn-Magee K. (2004). The Analytic Challenge in Interpretive Description. Int. J. Qual. Methods.

[28] Powell R.A., Single H.M. (1996). Focus Groups. Int. J. Qual. Health Care.

[29] Hennink M.M. (2013). Focus Group Discussions.

[30] Bradley E.H., Curry L.A., Devers K.J. (2007). Qualitative Data Analysis for Health Services Research: Developing Taxonomy, Themes, and Theory. Health Serv. Res..

[31] Hu T., Lee A.H. (2015). Commentary: Tobacco control and tobacco farming in African countries. J. Public Health Policy.

[32] Lecours N., Almeida G.E.G., Abdallah J.M., Novotny T.E. (2012). Environmental health impacts of tobacco farming: A review of the literature. Tob. Control.

[33] Glover D., Kusterer K. (2016). Small Farmers, Big Business: Contract Farming and Rural Development.

[34] Bellemare M.F. (2012). As You Sow, So Shall You Reap: The Welfare Impacts of Contract Farming. World Dev..

[35] Contract Farming as Partial Insurance. https://marcfbellemare.com/wordpress/wp-content/uploads/2017/07/BLNContractFarmingJuly2017.pdf.

[36] Otsuka K., Nakano Y., Takahashi K. (2016). Contract Farming in Developed and Developing Countries. Annu. Rev. Resour. Econ..

[37] Altman D.G., Levine D.W., Howard G., Hamilton H. (1996). Tobacco farmers and diversification: Opportunities and barriers. Tob. Control.

[38] Indonesia—Agribusiness Market and Support Activity (AMARTA). https://www.dai.com/our-work/projects/indonesia-agribusiness-market-and-support-activity-amarta.

[39] Markelova H., Meinzen-Dick R., Hellin J., Dohrn S. (2009). Collective action for smallholder market access. Food Policy.

[40] Ogundeji A.A., Onakuse S., Donkor E., Motsoari C. (2018). Impact of access to credit on farm income: Policy implications for rural agricultural development in Lesotho. Agrekon.

[41] Alemu A.E. (2017). Smallholders’ access to agricultural markets and technology, role of agricultural cooperatives and contracts in Africa—Evidence from dairy farmers in Ethiopia. Afr. Insight.

[42] Mitra S., Prodhan M.M.H. (2018). Factors Determining Credit Access of Tomato Farmers in A Selected Area of Bangladesh.

[43] Saqib S.E., Kuwornu J.K.M., Panezia S., Ali U. (2018). Factors determining subsistence farmers’ access to agricultural credit in flood-prone areas of Pakistan. Kasetsart J. Soc. Sci..

[44] Abimbola O.A. (2014). Post-harvest losses and welfare of tomato farmers in Ogbomosho, Osun state, Nigeria. J. Stored Prod. Postharvest Res..

[45] Rapsomanikis G. (2015). The Economic Lives of Smallholder Farmers. An Analysis Based on Household Data from Nine Countries.

[46] Magati P., Qing L., Drope J., Lencucha R., Labonte R. (2016). The Economics of Tobacco Farming in Kenya.

[47] Beach R.H., Jones A.S., Tooze J.A. (2008). Tobacco Farmer Interest and Success in Income Diversification. J. Agric. Appl. Econ..

[48] Narayanan S. (2012). Notional Contracts: The Moral Economy of Contract Farming Arrangements in India.

